# Intrinsically Stretchable Organic Electrochemical Transistors with Rigid‐Device‐Benchmarkable Performance

**DOI:** 10.1002/advs.202203418

**Published:** 2022-07-29

**Authors:** Dingyao Liu, Xinyu Tian, Jing Bai, Yan Wang, Yixun Cheng, Weijie Ning, Paddy K. L. Chan, Kai Wu, Junqi Sun, Shiming Zhang

**Affiliations:** ^1^ Department of Electrical and Electronic Engineering The University of Hong Kong Hong Kong SAR China; ^2^ Department of Mechanical Engineering The University of Hong Kong Hong Kong SAR China; ^3^ State Key Laboratory of Polymer Materials Engineering College of Polymer Science and Engineering Sichuan University Chengdu 610065 China; ^4^ State Key Laboratory of Supramolecular Structure and Materials College of Chemistry Jilin University Changchun 130012 China

**Keywords:** high performance, oxygen permeability, poly(3,4‐ethylenedioxythiophene):poly(styrene‐sulfonate), stretchable organic electrochemical transistors, stretchable substrates

## Abstract

Intrinsically stretchable organic electrochemical transistors (OECTs) are being pursued as the next‐generation tissue‐like bioelectronic technologies to improve the interfacing with the soft human body. However, the performance of current intrinsically stretchable OECTs is far inferior to their rigid counterparts. In this work, for the first time, the authors report intrinsically stretchable OECTs with overall performance benchmarkable to conventional rigid devices. In particular, oxygen level in the stretchable substrate is revealed to have a significant impact on the on/off ratio. By employing stretchable substrates with low oxygen permeabilities, the on/off ratio is elevated from ≈10 to a record‐high value of ≈10^4^, which is on par with a rigid OECT. The device remained functional after cyclic stretching tests. This work demonstrates that intrinsically stretchable OECTs have the potential to serve as a new building block for emerging soft bioelectronic applications such as electronic skin, soft implantables, and soft neuromorphic computing.

## Graphical abstract

It is observed that the poor electrical performance of stretchable organic electrochemical transistors (OECTs) can be significantly improved by controlling the oxygen permeability of the elastic substrate. The overall performance of the optimized device is verified benchmarkable to a conventional rigid OECT.

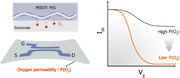

## 1 Introduction

Organic electrochemical transistors (OECTs) based on poly(3,4‐ethylenedioxythiophene): poly(styrene‐sulfonate) (PEDOT:PSS) have emerged as powerful transducers for bioelectronic applications due to their high transconductance,^[^ ^1^, ^2^, ^3^, ^4^ ^]^ water stability,^[^ ^5^, ^6^, ^7^, ^8^ ^]^ and ion‐to‐electron converting ability at low voltages.^[^ ^9^, ^10^ ^]^ OECTs hold the world‐record sensitivity in detecting brain signals.^[^ ^11^, ^12^ ^]^ Moving forward, stretchable OECTs are needed to minimize their mechanical mismatch with soft biological objects to reduce motion artifacts.^[^ ^13^, ^14^, ^15^ ^]^ For example, when used as skin‐attachable active electrodes, OECTs should be able to withstand a strain of at least 30% to conform to skin deformation.^[^ ^16^, ^17^, ^18^, ^19^, ^20^, ^21^ ^]^ Therefore, pioneer works have been dedicated to developing stretchable OECTs. A major challenge for the development of stretchable OECTs is that the material systems and fabrication methods need to be systemically rebuilt.^[^ ^22^, ^23^ ^]^ In 2017, Zhang et al. reported the first fully stretchable OECT on elastic polydimethylsiloxane (PDMS) substrate.^[^ ^13^ ^]^ The device was fabricated by using the buckling method and a solid‐state hydrogel as the stretchable electrolyte. A 10*10 stretchable OECT microarray was demonstrated. The stretchable OECTs could withstand a strain up to 30% with stable performance. In 2018, Ramuz et al. reported stretchable OECTs by using the laser etching method to pattern stretchable serpentine‐shaped electrodes and channels.^[^ ^24^ ^]^ The device could maintain a high transconductance of 0.35 mS at 38% strain. In 2018, Lee et al. fabricated stretchable OECTs with a stretchable nano‐mesh architecture. The device was used for conformal electrocardiogram recording in a living rat.^[^ ^14^ ^]^ In 2019, Zhang et al. reported the first intrinsically stretchable OECTs by using ultrathin and microcracked gold film as stretchable interconnect and microcracked PEDOT:PSS film as stretchable channel.^[^ ^25^ ^]^ Afterward, Matsuhisa et al. reported the use of intrinsically stretchable OECTs for the demonstration of stretchable synaptic transistors.^[^ ^26^ ^]^ In 2019, Li et al. reported stretchable OECTs for glucose detection. The stretchable OECTs sensor remained functional between 0% and 30% strain.^[^ ^27^ ^]^ In 2021, Nguyen et al. demonstrated an artificial synapse with stretchable OECT, where the synaptic behavior could be controlled by regulating the dynamics of ion transport.^[^ ^28^ ^]^ In 2022, new attempts were reported to fabricate stretchable OECTs with newly synthesized conducting polymers.^[^ ^29^, ^30^ ^]^

Despite the above achievements in advancing stretchable OECTs, the performance of intrinsically stretchable OECTs remains much lower compared to their rigid counterparts. For example, the on/off ratio of intrinsically stretchable PEDOT:PSS OECTs fabricated on stretchable substrates such as PDMS was two orders of magnitude lower than that of a rigid device.^[^ ^13^, ^25^, ^26^, ^31^ ^]^ Besides, the charge carrier mobility of stretchable OECTs has yet to be investigated. The on/off ratio and mobility of stretchable OECTs are essential parameters for evaluating their frequency response and switching properties for emerging soft bioelectronic applications such as stretchable neuroelectronic computing^[^ ^32^ ^]^ and stretchable display.^[^ ^33^ ^]^ Therefore, in the past years, intensive efforts have been dedicated to resolving these problems, including using a buffer layer between the substrate and channel ^[^ ^25^ ^]^ and using different substrates.^[^ ^34^ ^]^ However, limited progress was made, and the underlying reasons remain unclear.

In this work, we report the first intrinsically stretchable PEDOT:PSS OECTs with overall performance benchmarkable to a rigid device. In particular, we reveal that the oxygen level of the stretchable substrates plays a decisive role in affecting the on/off ratio. By employing stretchable substrates with low oxygen permeability, the on/off ratio was elevated from ≈10 to a record‐high value of ≈10^4^. The device remained functional after cyclic strain tests between 0% and 50%. Besides, those devices showed high mobilities of ≈1.1 cm^2^ V^−1^ s^−1^, comparable to that of the rigid device. These results encourage further development of intrinsically stretchable OECTs for emerging soft bioelectronics applications.

## 2 Results and Discussion

**Figure 1:**
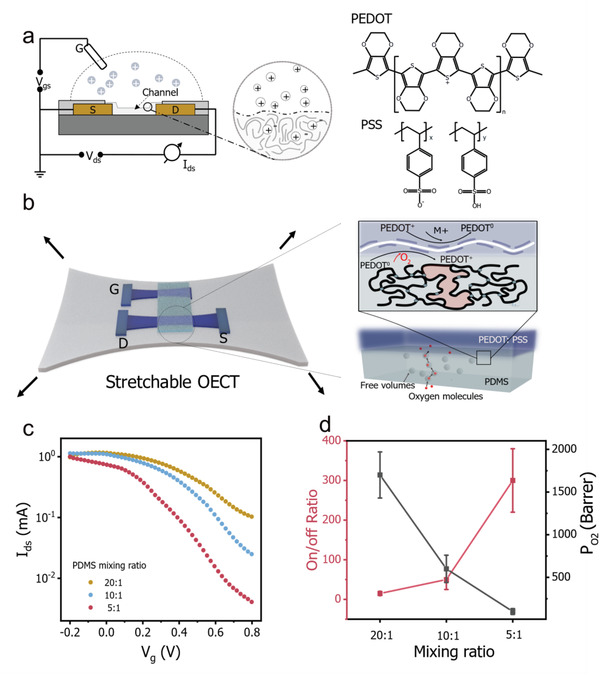
Performance of intrinsically stretchable OECTs on substrates with different oxygen permeabilities (*P*_O2_). a) Structure of OECTs based on conducting polymer PEDOT:PSS channel. b) The schematic of an intrinsically stretchable OECT. Stretchable elastomers tend to have a larger free volume, which increases the oxygen level and prevents PEDOT^+^ from de‐doping at the substrate/channel interface. c) Transfer curves of intrinsically stretchable OECTs fabricated on PDMS substrates of different mixing ratios. d) The correlation between on/off ratios of the devices and oxygen permeabilities (*P*_O2_) of the substrates.

Compared to a rigid device, PEDOT:PSS OECTs fabricated on stretchable elastomers have been suffering from low on/off ratios. Given that the on/off ratio of OECT is mainly dominated by the off‐state current (i.e., the de‐doping level of the PEDOT:PSS channel) (**Figure** **1**a), we thus hypothesized that there are hidden parameters in the stretchable substrate preventing the channel from de‐doping. We further hypothesized oxygen permeability (*P*_O2_) is a dominant hidden parameter. As is known, oxygen molecules significantly affect the redox process.^[^ ^35^, ^36^, ^37^ ^]^ Different from the rigid substrates, stretchable elastomers are porous and have large free volumes, which can provide accessible pathways for oxygen molecules (Figure 1b). It's worth noting that the oxygen level in some stretchable elastomers can be ten times higher than that in the electrolyte^[^ ^38^, ^39^ ^]^ thus, it may considerably affect the on/off ratio.

To verify the above hypothesis, we prepared PDMS elastomers with different P_O2_ values by controlling the mixing ratios.^[^ ^40^ ^]^ Stretchable OECTs were subsequently fabricated on these substrates (Figure 1c, detailed in Experimental Section). The correlation between the on/off ratios and the *P*_O2_ values is shown in Figure 1d. In line with our hypothesis, reducing the *P*_O2_ value significantly reduces the off‐state current (Figure 1c). For example, an off‐state current of 10^−1^ mA was measured on substrates with *P*_O2_ of ≈1700 Barrer, while the value dropped to 5*10^−3^ mA when the *P*_O2_ value decreased to ≈100 Barrer (Figure 1c). As a result, the on/off ratio increased dramatically from 10 to 300 (Figure 1d) ((*I*_ds_ (*V*_g_ = 0 V)/*I*_ds_ (*V*_g_ = 0.8 V)).

**Figure 2:**
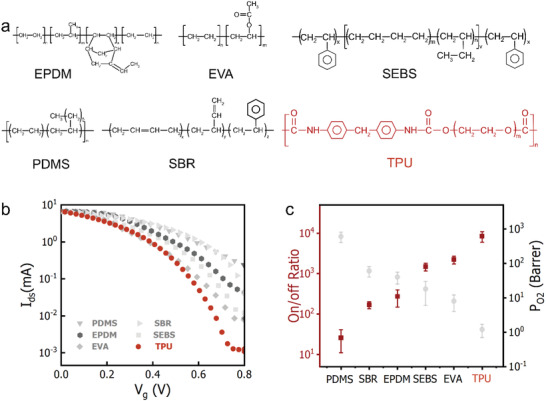
Comparison of on/off ratios of stretchable OECTs fabricated on different types of stretchable substrates with different *P*_O2_ values. a) The chemical structures of different stretchable substrates. b) Transfer curves of stretchable OECTs fabricated on various substrates. c) The correlation between the on/off ratios of devices and the *P*_O2_ values of corresponding substrates.

To further verify the above results, we subsequently fabricated devices on different types of common elastomers, including PDMS, styrene‐butadiene rubber (SBR), ethylene propylene diene monomer rubber (EPDM), poly(styrene‐ethylene‐butylene‐styrene) (SEBS), ethylene‐vinyl acrylate (EVA) and thermoplastic polyurethane (TPU) (**Figure** **2**a). The fabrication condition was controlled to let *P*_O2_ be the major variable parameter (See details in Experimental Section). The correlation between the on/off ratios and the *P*_O2_ values of those different substrates is shown in Figure 2b. In agreement with the above results, an inverse relationship was found between the *P*_O2_ values and the on/off ratios. That is, OECTs on substrates with lower *P*_O2_ values obtained higher on/off ratios. In particular, the device fabricated on the TPU substrate with an extremely low *P*_O2_ value of ≈1.0 Barrer obtained a record‐high on/off ratio of ≈10^4^, which is benchmarkable to the best value reported for a rigid device of the same dimension. These results were further verified by comparing on/off ratios of devices fabricated on TPU substrates with different *P*_O2_ values (Figure S1, Supporting Information).

**Figure 3:**
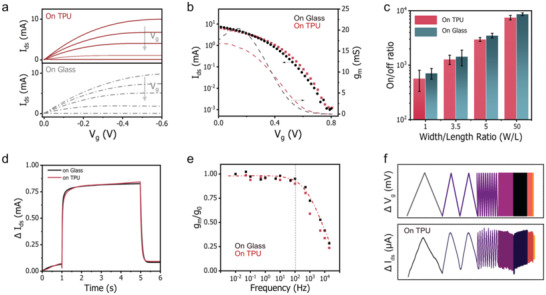
Benchmarking the overall performance of intrinsically stretchable OECTs (on low *P*_O2_ substrates) to rigid OECTs (on glass). a) Output curves. The *V*_ds_ is scanned from 0 V to −0.6 V. The *V*_g_ is swept from 0 to 0.8 V, with a step of 0.2 V. b) Transfer curves. The *V*_ds_ is fixed at −0.4 V. The *V*_g_ is swept from 0 V to 0.8 V. c) The on/off ratios of intrinsically stretchable OECTs and rigid OECTs with different geometries (*W*/*L*). Detailed transfer curves are shown in Figure S4, Supporting Information. d) Transient response curves. A pulse of V_g_ of 0.4 V is applied, and the *V*_ds_ is fixed at −0.2 V. e) The frequency response profiles of the *g*_m_. The *V*_ds_ is fixed at −0.4 V, and a small signal oscillation of 20 mV is superimposed on a constant *V*_g_ of 0.1 V to measure the transient *g*_m_.^[^ ^4^ ^]^ f) The output *I*_ds_ of intrinsically stretchable OECT in response to a triangle wave input from 0.05 to 10^4^ Hz.

The high on/off ratio obtained on stretchable substrates of low *P*_O2_ values (such as TPU) encourages us to compare its overall performance with a rigid device. To do so, we first compared their steady‐state performance, including the output and transfer curves. These devices were designed of the same dimension and used the same electrolyte to let the substrate be the major variable parameter. The results are summarized in **Figure** **3**a,b. As shown, both devices showed similar output and transfer profiles. From the transfer curves, we extracted the corresponding on/off ratios. As shown in Figure 3c and Figure S2 in Supporting Information, both devices showed comparable on/off ratios. The conclusion is appliable to devices with other *W*/*L* ratios. The on/off ratio reached a high value of ≈10^4^ when the *W*/*L* ratio was increased to 50. We further extracted a transconductance (*g*_m_) of 15 mS (at *V*_g_ = 0.1 V) from those stretchable devices. To the best of our knowledge, this is the highest *g*_m_ reported so far for intrinsically stretchable PEDOT:PSS OECTs.

Next, we compared the transient behavior of those intrinsically stretchable OECTs and rigid OECTs, including their transient response and frequency response. We obtained similar transient response (Figure 3d) and frequency response (Figure 3e) curves for both devices (*W*/*L* = 50). The *g*_m_ value maintained stable up to 100 Hz, comparable to the rigid device of the same geometry.^[^ ^41^ ^]^ Besides, the extracted mobility (detailed in Figures S3‐S4, Supporting Information) was as high as ≈1.1 cm^2^ V^−1^ s^−1^, again comparable to the rigid device's value (≈1.2 cm^2^ V^−1^ s^−1^).

**Figure 4:**
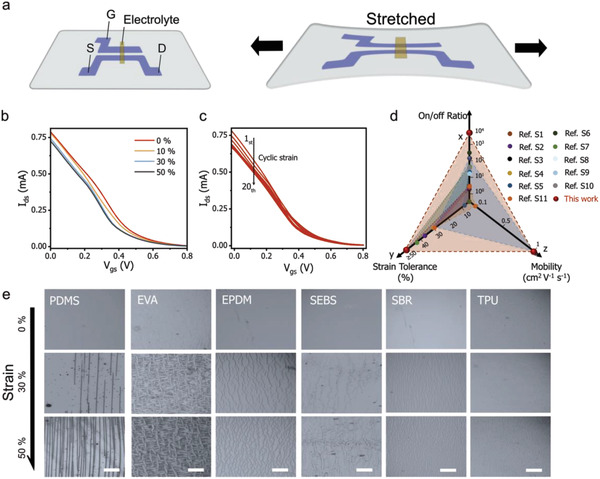
Performance of intrinsically stretchable OECTs under strain. a) The architecture of the intrinsically stretchable OECTs. PEDOT:PSS films are used as both the electrodes (source, drain, and gate) and the channel. b) Transfer curves at different strain values. The *V*_g_ is swept from 0 to 0.8 V, and *V*_ds_ is fixed at −0.4 V. c) Transfer curves after different strain cycles (50% strain). d) Overall performance comparison of the intrinsically stretchable OECTs in this work with previously reported stretchable OECTs. The x‐axel indicates the highest on/off ratios, the y‐axel indicates the maximum tolerable strains, and the z‐axel indicates the maximum hole mobilities of the channel. e) Optical microscopic images of PEDOT:PSS films fabricated on different stretchable substrates and under different strain values (0%, 30%, and 50%, respectively).

After confirming the high performance, we investigated the stretchability of devices. To make the device fully stretchable, stretchable PEDOT:PSS thin film was used as the planar gate electrode (**Figure** **4**a).^[^ ^42^ ^]^ A stretchable ionic gel was used as the solid‐state electrolyte, and liquid metal (eutectic gallium–indium, EGaIn) was used as probing electrodes to facilitate the connection (detailed in the Experimental Section and Figure S5, Supporting Information).^[^ ^13^ ^]^ Before the strain test, the device was pre‐stretched to 50% strain to stabilize the channel's conductance (Figure S6, Supporting Information). The transfer curves obtained during the strain test are shown in Figure 4b,c. Overall, these transfer curves showed minor changes at different strain values or increased strain cycles (Figure S7, Supporting Information). The same trend was recorded for the *g*_m_ (Figure S8, Supporting Information), demonstrating the robustness of the device. The mobility maintained at a stable value of ≈1 cm^2^ V^−1^s^−1^ before 15% strain. It slightly decreased to 0.85 cm^2^ V^−1^ s^−1^ at 50% strain (Figure S9, Supporting Information), attributable to the strain‐induced separation of the conductive PEDOT^+^ conjugated polymer chains,^[^ ^25^ ^]^ which prohibited the interchain hopping of the holes.^[^ ^43^, ^44^, ^45^ ^]^ The overall performance of the stretchable OECTs is summarized in Figure 4d and Table S1, Supporting Information.^[^ ^13^, ^21^, ^24^, ^25^, ^26^, ^28^, ^46^, ^47^, ^48^, ^49^ ^]^ To conclude, the device showed record‐high on/off ratio (≈10^4^), high mobility (≈1 cm^2^ V^−1^ s^−1^), and intrinsically stretchability (>50%). This is the first time these merits can be harvested in one intrinsically stretchable OECT, encouraging its immediate use for various soft bioelectronics applications.

Finally, we investigated the mechanism for the devices' high intrinsic stretchability by comparing the optical microscopic images of PEDOT:PSS films at different strain values (Figure 4e). We noted that PEDOT:PSS films on TPU substrates showed little cracks and maintained a substantial current at 50% strain, while films on other substrates showed evident cracking and a considerable current loss at lower strain values (Figure S10, Supporting Information). It is worth mentioning that the high intrinsic stretchability of PEDOT:PSS films on TPU was obtained without the addition of any plasticizers which were needed for previously reported works.^[^ ^50^ ^]^ Moreover, the film's high stretchability could be maintained after increasing the film's thickness to 1 µm (Figure S11, Supporting Information) or adding the (3‐glycidyloxypropyl) trimethoxy silane (GOPS) crosslinker (Figure S12, Supporting Information).^[^ ^51^, ^52^ ^]^ Besides, these films showed minor current loss under cyclic strain tests (Figure S13, Supporting Information). The high intrinsic stretchability is attributed to the improved matching of Young's modulus between the TPU substrates and the PEDOT:PSS films.^[^ ^53^, ^54^ ^]^ This conclusion is supported by the finite element analysis (Figured S14 and S15, Supporting Information), which demonstrates that an improved modulus match of those two layers facilitates the dissipation of the stress and favors the formation of microcracks rather than the large transverse cracks, thus improving the intrinsic stretchability of the channel.

## 3 Conclusions

In summary, we have reported the first intrinsically stretchable PEDOT:PSS OECTs with overall performance benchmarkable to a rigid device. The device obtained a record‐high on/off ratio of ≈10^4^, high mobility of ≈1.1 cm^2^·V^−1^·s^−1^, and maintained stable performance up to 50% strain. The high performance was realized by minimizing the *P*_O2_ values of the stretchable substrates, which facilities the de‐doping of the PEDOT:PSS channel. The presented high‐performance intrinsically stretchable OECTs can be immediately used as a new device paradigm to impact the field of soft bioelectronics. We envision that this work will significantly promote the further development of of tissue‐like OECTs in those fast‐rising research areas such as epidermal biosensing,^[^ ^55^, ^56^ ^]^ soft neuromorphic computing,^[^ ^57^ ^]^ and soft human‐machine interfaces.^[^ ^58^ ^]^

## 4 Experimental Section

### Materials

PEDOT:PSS aqueous suspension (Clevios PH1000) was purchased from Heraeus Electronic Material (USA). Glycerol, dodecylbenzene sulfonic acid (DBSA), sodium chloride, (3‐glycidyloxypropyl) trimethoxysilane (GOPS), cetyltrimethylammonium bromide (CTAB), ammonium persulfide (APS), acrylamide (AAm), *N*′‐Tetramethylethylenediamine (TEMED) and *N*,*N*′‐methylenebisacrylamide (MBAA) were purchased from Sigma‐Aldrich Co (USA). The liquid metal, eutectic gallium‐indium (EGaIn), was supplied by Flystone Electronics Co. (Zhejiang, China). PDMS (Sylgard 184 silicone elastomer) was purchased from Dow Corning (USA). Styrene‐butadiene rubber (SBR) was provided by Baling Petrochemical Co (China). Ethylene‐vinyl acetate copolymer (EVA50, 50% vinyl acetate) was purchased from Yanshan Petrochemical Co. (China). Polyether‐based TPU was provided by Covestro AG. (Desmopan 9385) and BASF Co. (Elastollan 1195 A). The semi‐permeable TPU film was purchased from 3M Co. (The United States). Poly(styrene‐ethylene‐butylene‐styrene) (SEBS) was provided by Sigma‐Aldrich Co. (The United States). *N*, *N*‐dimethylformamide (DMF), tetrahydrofuran (THF), and toluene were obtained from Aladdin Co. (Shanghai, China). The silver paste was purchased from Voltera Co. (USA). The water‐soluble tape (HD 5414) was provided by 3M Co. (USA).

### Preparation of PEDOT:PSS Mixtures

PEDOT:PSS aqueous suspension was firstly stirred for 3 min and then mixed with glycerol (5 v/v.%) and DBSA (0.1 v/v.%) with a Vortex (MX‐S). The addition of glycerol was to increase the film conductivity. DBSA was added to facilitate the wetting property of films on substrates. Then the mixed suspension was filtered with a polytetrafluoroethylene (PTFE) membrane (aperture size of 0.45 µm) to remove aggregates for further use.

### Preparation of Stretchable Substrates

Elastic substrates were fabricated through a typical solution casting process. Under the heating temperature of 80 °C, TPU grains were dissolved in DMF, SEBS and SBR were dissolved in toluene. EVA grains were dissolved in THF at room temperature. For all these elastomer solutions, 10 w/w.% solutions were prepared by mixing 1 g elastomer with 9 g corresponding solvent. After being fully dissolved, the obtained solution was cast on a 2.5 cm * 7.5 cm glass slide, and the elastic substrates were obtained after drying overnight at room temperature in the fume hood. Before preparing PDMS substrates, CTAB solution (0.005 M) was prepared and then spun coated on a glass slide as an anti‐adhesive layer to ease PDMS peeling‐off at the end of the process. In the next, the base and curing agent of PDMS were mixed. To obtain PDMS with different oxygen permeability, the mixing ratio of the base and curing agent was controlled between 5:1 (low permeable) and 20:1 (highly permeable).^[^ ^59^ ^]^ After removing the bubbles under vacuum, the premixed slurry was spin‐coated on the glass substrates at 500 rpm for 10 s and 1000 rpm for 30 s. Afterward, the samples were cured at 80 °C for 30 min in an oven. Finally, the PDMS substrates were detached from the glass slides for future use.

### Synthesis of Stretchable Gel

First, the powder of 15.5 w/w.% AAm was dissolved in 16 mL of deionized water. Then, 0.01875 w/w% MBAA was added as the crosslinker for polyacrylamide. 1.2 w/w.% APS was added as a photo‐initiator for polymerization. 10 w/w.% NaCl was added to improve the ionic conductivity. Then, TEMED was added to the above mixtures as the crosslinking accelerator at the 0.025% weight of AAm. Afterward, the precursor solution was transferred to a petri dish and cured by exposure to an ultraviolet lamp (365 nm, 5 mW·cm^−2^; Spectroline). Then the non‐volatile stretchable gel can be obtained after solvent exchange by immersing the cured hydrogel into a bath composed of 85 w/w.% glycerol, 5 w/w.% water, and 10 w/w.% NaCl for 2 days.

### Fabrication of Stretchable OECT

When benchmarking the performance of OECT on different substrates, the OECT was fabricated firstly by patterning source and drain electrodes, followed by a baking process under 120 °C. Subsequently, a layer of PDMS was pasted on the top of the source and drain electrodes for insulation. On different substrates, PEDOT:PSS may have different film‐forming capabilities. To avoid such an effect, PEDOT:PSS film was prepared by firstly spin‐coating the mixture suspension on glass slides and then transferred to targeted substrates with a water‐soluble tape. To confine the electrolyte, a well was then defined on the top of the channel with a hollow cylinder (diameter of 10 mm). Then a commercial Ag/AgCl electrode was used as gate electrode, and a 0.1 M NaCl solution was filled in the well as an electrolyte. For stretchable OECT devices, PEDOT:PSS film was used as the planar gate electrode aside the channel. Two parallel PEDOT:PSS stripes were patterned onto the TPU substrate by using a shadow mask. The shadow mask was made of polyamide tape by laser cutting. Firstly, the mask was attached to the TPU substrate, and then PEDOT:PSS suspension was spun‐coated (500 rpm for 15 s and then 1500 rpm for 45 s) on top of the surface. After lifting off the shadow mask, a patterned PEDOT:PSS channel was obtained after baking the film at 100 °C for 20 min. Then, the solid‐state gel was used as a stretchable electrolyte by casting on the channel and gate. EGaIn was used to facilitate the probing of the stretchable electrodes for the strain test.

### Characterizations

Film thickness measurements were performed with a step profilometer (Bruker, Dektak XT). The electrical performance of PEDOT:PSS films was measured using Agilent B2900A controlled with LabVIEW software. The strain test of the film was performed with a tensile machine (Feinixs, FMSXX 80‐50‐50). For transistor characterizations, the transfer, output, and transient responses were measured with Agilent B2902A source‐meter unit controlled with Quick IV software. The cracking of PEDOT:PSS film under strain was studied with a Nikon optical microscope. The Young's modulus of the elastomers was measured with an Instron E‐3000 tensile tester under a slow tensile rate of 5 mm min^−1^. The O_2_ permeation analysis was conducted under constant volume/variable pressure conditions using a VAC‐V2 film permeability testing machine (Labthink Instruments, Jinan, China).

## Conflict of Interest

The authors declare no conflict of interest.

## Supporting information

Supporting Information

## Data Availability

The data that support the findings of this study are available from the corresponding author upon reasonable request.
