# Supplementary material for: Intrinsically Stretchable Organic Electrochemical Transistors with Rigid‐Device‐Benchmarkable Performance
Source: Adv Sci (Weinh). 2022 Jul 29;9(29):2203418. doi: 10.1002/advs.202203418 (PMC9561867; doi:10.1002/advs.202203418)
Supplement: Supplementary file 1 — Supporting Information [file ADVS-9-2203418-s001.pdf]

ADVANCED SCIENCE
Open Access

Supporting Information

for *Adv. Sci.*, DOI 10.1002/advs.202203418

Intrinsically Stretchable Organic Electrochemical Transistors with Rigid-Device-Benchmarkable Performance

*Dingyao Liu, Xinyu Tian, Jing Bai, Yan Wang, Yixun Cheng, Weijie Ning, Paddy K. L. Chan, Kai Wu, Junqi Sun and Shiming Zhang**

Supporting information

Intrinsically Stretchable Organic Electrochemical Transistors with Rigid-Device-Benchmarkable Performance

*Dingyao Liu[1], Xinyu Tian[1], Jing Bai[1], Yan Wang[1], Yixun Cheng[1], Weijie Ning[1], Paddy K. L. Chan[2], Kai Wu[3], Junqi Sun[4], Shiming Zhang[1]**

[1]Department of Electrical and Electronic Engineering, The University of Hong Kong, Hong Kong SAR, China

[2]Department of Mechanical Engineering, The University of Hong Kong, Hong Kong SAR, China

[3]State Key Laboratory of Polymer Materials Engineering, College of Polymer Science and Engineering, Sichuan University, Chengdu 610065, China

[4]State Key Laboratory of Supramolecular Structure and Materials, College of Chemistry, Jilin University, Changchun 130012, China

E-mail: szhang@eee.hku.hk

This file includes:

Figure S1. The on/off ratios of OECT device on different types of TPU.
Figure S2. The transfer curves of rigid and stretchable OECTs with different geometries (W/L).
Figure S3. Comparison of mobility of PEDOT: PSS channel on glass and low Po_2 TPU substrates.
Figure S4. Comparison of mobility of PEDOT: PSS channel on PDMS and TPU substrates.
Figure S5. Transfer and output curves of fully PEDOT: PSS-based stretchable OECT.
Figure S6. The transfer curves of stretchable OECTs during the pre-stretching process.
Figure S7. The strain-insensitive feature of the stretchable OECT.
Figure S8. The change of g_m for stretchable OECT under strain.
Figure S9. Comparison of the channel mobility at different strain values.
Figure S10. Comparison of the resistance change (R/R_0) of the PEDOT: PSS films on different substrates.
Figure S11. Thickness-dependent stretchability profile of PEDOT: PSS films on TPU substrate.
Figure S12. The water stability of PEDOT: PSS films on TPU with and without the addition of GOPS.
Figure S13. The cyclic stability of the electromechanical performance of PEDOT: PSS film on TPU.
Figure S14. Mechanical profiles of TPU and PDMS elastomers.
Figure S15. Finite element analysis of the stress distribution for PEDOT: PSS films on different substrates.
Table S1. Benchmarking the performance of stretchable OECTs reported so far.

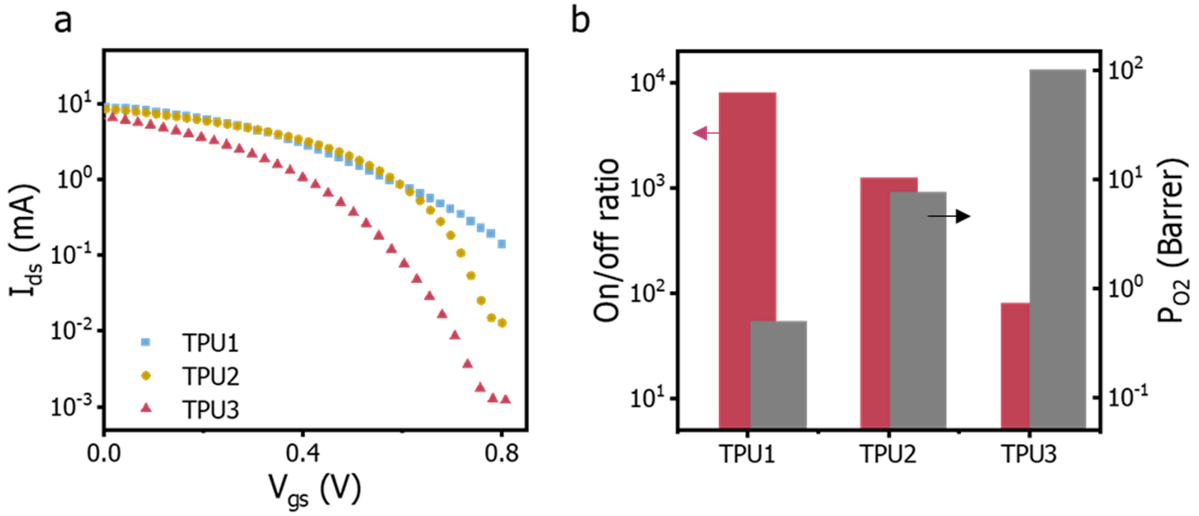

Figure S1. The on/off ratios of OECT device on different types of TPU. (a) The transfer curves obtained from OECT fabricated on three different types of TPU substrates (V_{ds} = -0.4 V). TPU1 (Elastollan® 1195 A) was provided by BASF TPU with tightly pack segments. TPU2 is a polyether-based elastomer provided by Covestro AG (Desmopan® 9385). TPU3 is a semi-permeable TPU film (3M Tegaderm). (b) The on/off ratios of devices fabricated on three types of TPU substrates and the corresponding oxygen permeability (P_{O2}) of those substrates.

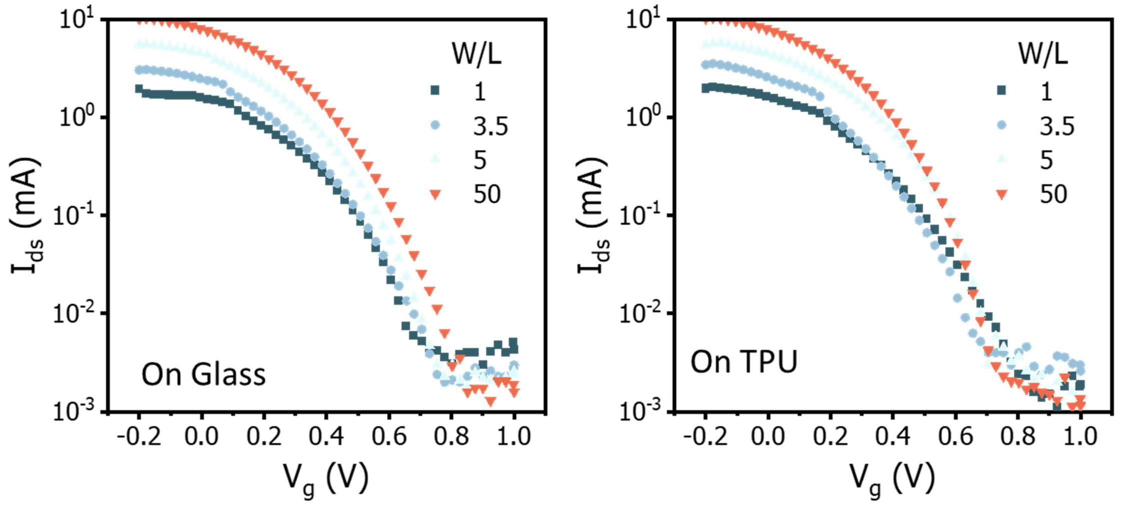

Figure S2. The transfer curves of rigid (left) and stretchable (right) OECTs with different geometries (W/L). The V_{ds} was -0.4 V, the width (W) of the devices was fixed at 5 mm.

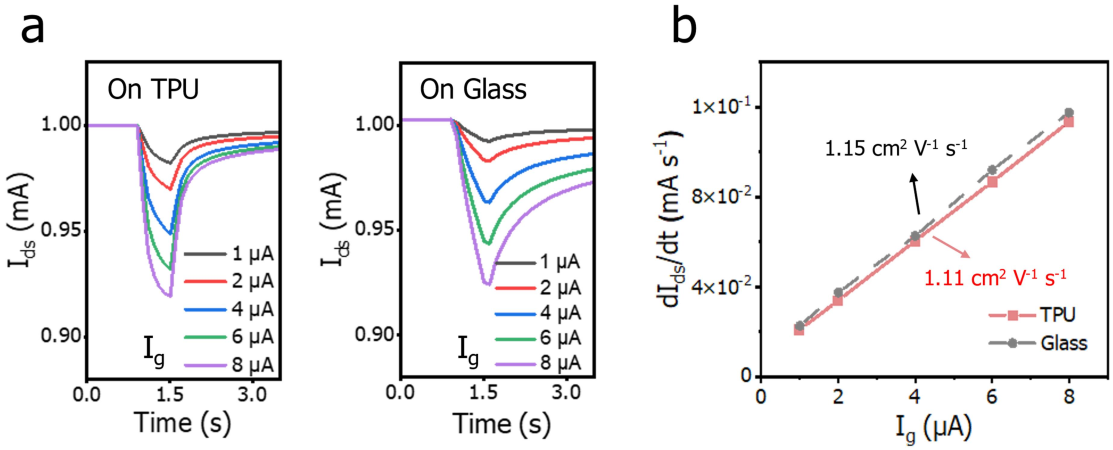

Figure S3. Comparison of mobility of PEDOT: PSS channel on glass and low Po_2 TPU substrates. (a) The response of I_{ds} on TPU substrate (left) and on glass substrate (right), at different gate current (I_{gs}) values. A constant V_{ds} of -0.2 V was applied. The channel width (W) is 5 mm, and the channel length (L) is 1.3 mm. (b) Derivative of I_{ds} with respect to time as a function of I_{gs}.

According to the Malliaras-Bernards model,[1] the response of OECT can be understood by considering the OECT consisting of two circuits: the ionic one, where ions are transported between the electrolyte and the channel, and the electronic one where holes are transported in the PEDOT: PSS channel between the source and drain. The ionic circuit is governed by the species of ions and the interface between electrolyte and channel. The latter can be estimated by driving the OECT with constant gate current and simultaneously measuring the change of source-drain current regarding response time, and the mobility of PEDOT: PSS on both soft and rigid substrate can be calculated via Eq. (1) to Eq. (3):

$$I_{ds}(t, I_{gs}) = I_{ds0} - I_{gs}(f + \frac{t}{\tau_e}) \quad (1)$$

$$\frac{dI_{ds}}{dI_t} = -\frac{I_{gs}}{\tau_e} \quad (2)$$

$$\tau_e = -L^2/uV_{ds} \quad (3)$$

where t is the time, I_{ds} is the source-drain current before application of I_{gs}, f is a proportionality constant to account for the spatial non-uniformity of the de-doping process, τ_e is the electronic transit time, L is the channel length of OECT (1.3 mm) and V_{ds} is the source-drain voltage (-0.2 V). u denotes the hole mobility of PEDOT: PSS channel. According to Eq. (1) and (2), we could calculate the electronic transit time from the linear transient response range of I_{ds} in Figure S1a. Subsequently, we could extract the mobility (u) according to Eq. (3).

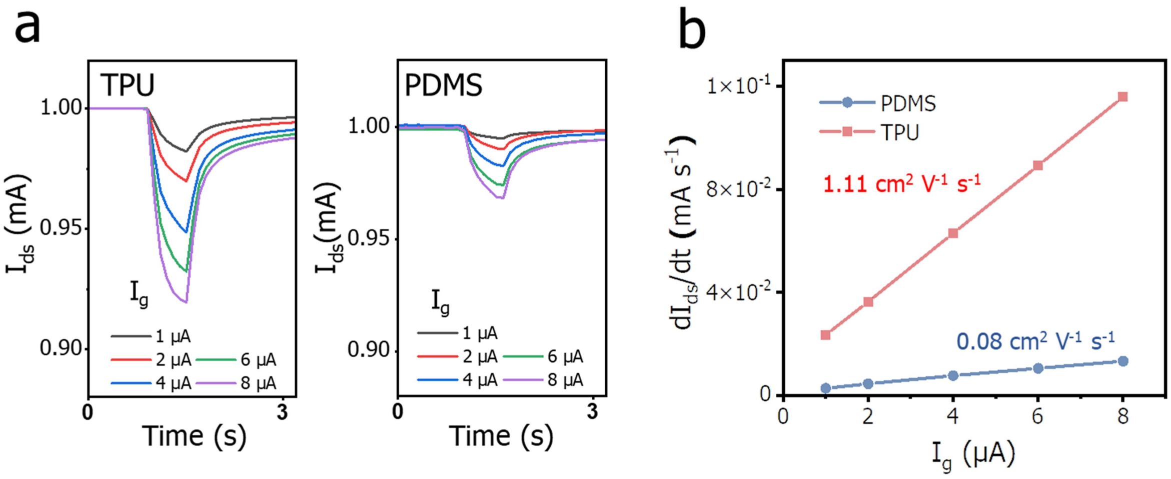

Figure S4. Comparison of mobility of PEDOT: PSS channel on PDMS and TPU substrate. (a) The response of I_{ds} when a series of I_{gs} was applied to stretchable OECT on TPU (left) and PDMS (right). A constant V_{ds} of -0.2 V was applied. The device has a channel width (W) of 5 mm and channel length (L) of 1.3 mm. (b) Derivative of I_{ds} with respect to time as a function of I_{gs}.

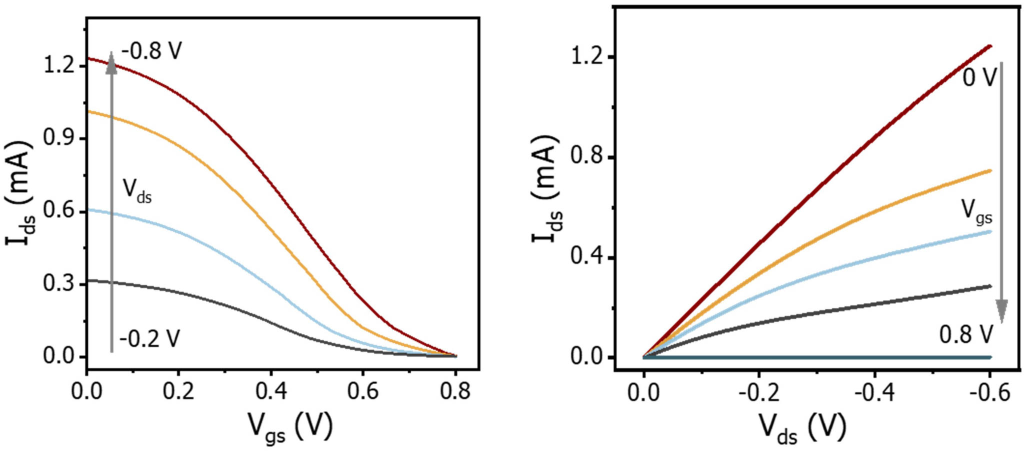

Figure S5. Transfer (left) and output (right) curves of fully PEDOT: PSS based stretchable OECT.

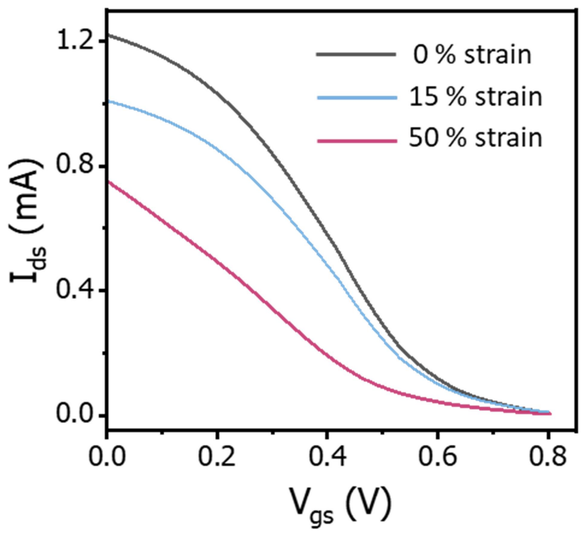

Figure S6. The transfer curves of stretchable OECT at different strain values. The fully stretchable OECT was fabricated on TPU. A solid-state and stretchable gel was used as the stretchable electrolyte. Liquid metal (EGaIn) was used to facilitate the probing of the electrodes during the strain test.

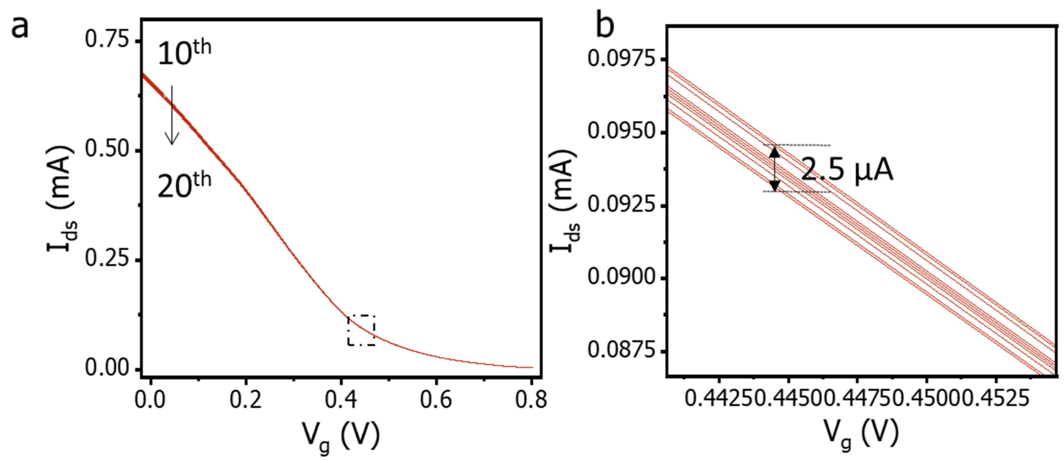

Figure S7. The strain-insensitive feature of the stretchable OECT. (a) The transfer curves of the stretchable OECT device being stretched among the 10th to 20th circles and **(b)** their zoom-in figure.

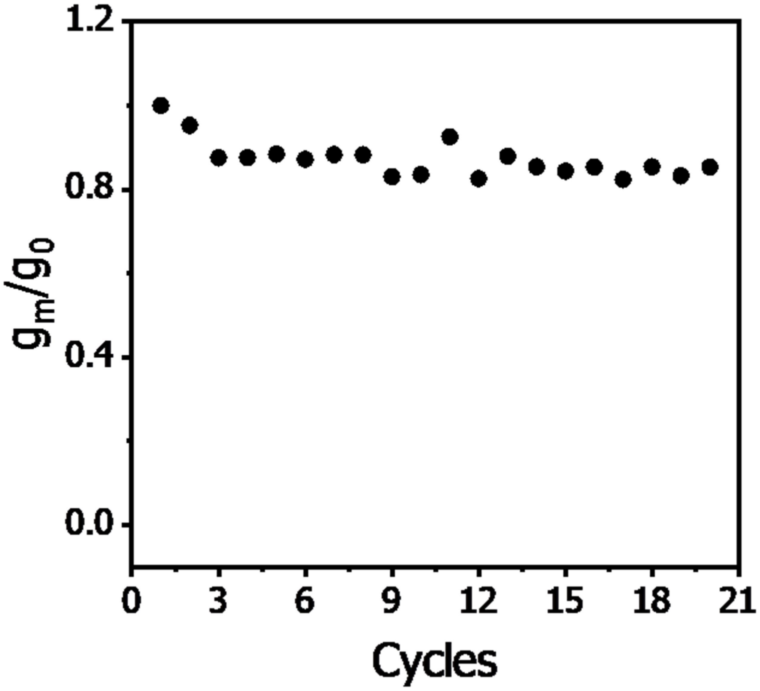

Figure S8. The change of maximum transconductance (g_m) of stretchable OECT after multiple strain (50%) cycles.

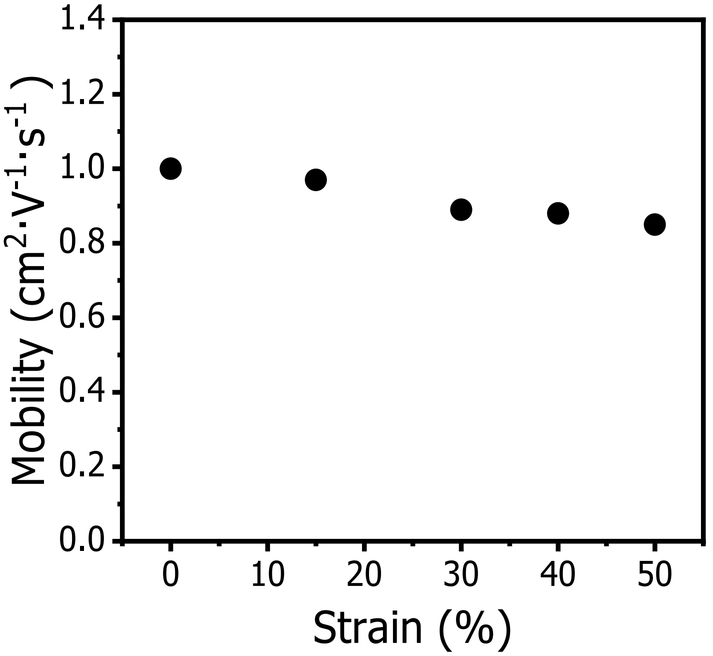

Figure S9. Comparison of the channel mobility at different strain values.

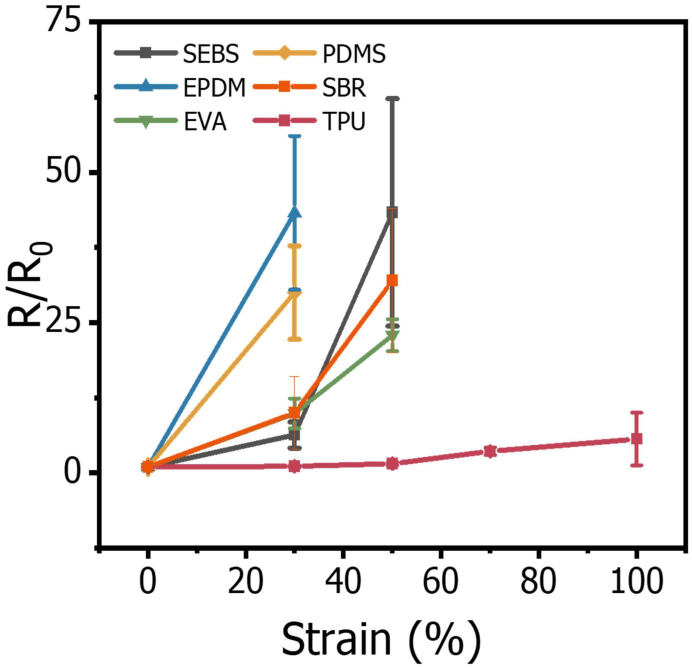

Figure S10. Comparison of the resistance change (R/R_0) of the PEDOT: PSS films fabricated on different stretchable substrates.

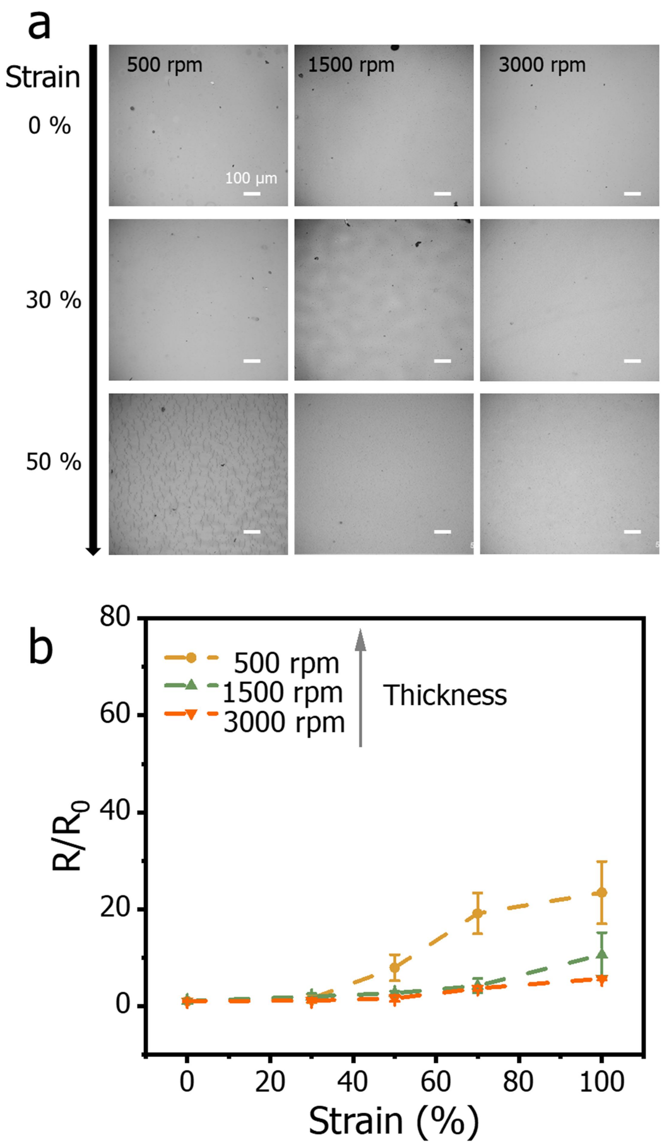

Figure S11. Thickness-dependent stretchability profile of PEDOT: PSS films on TPU substrates. (a) The optical images of PEDOT: PSS films of different thicknesses under different strain values (0%, 30%, and 50 % respectively). Note that the thickness was controlled by changing the spin-coating speed (from 500*30 s rpm to 3000*30 s rpm). **(b)** Strain-resistance profile of PEDOT: PSS films with different thicknesses. The dimension of the PEDOT: PSS films used were 1 cm in length and 0.5 cm in width. The resistance was measured by using liquid metal as the probing electrode.

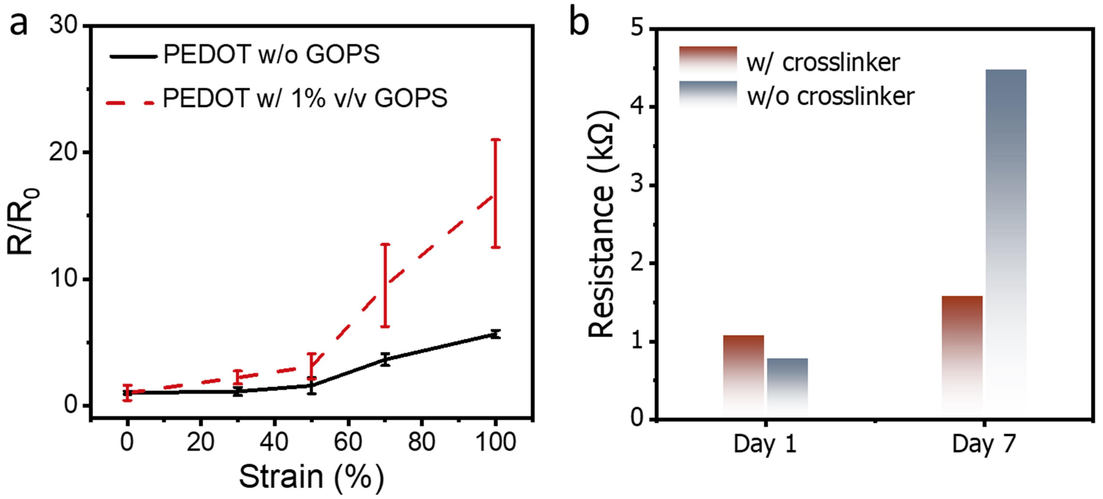

Figure S12. The water stability of PEDOT: PSS films on TPU with and without the addition of GOPS. (a) Comparison of the resistance change under strain for PEDOT: PSS film before and after adding GOPS. (b) Enhanced water stability was obtained by the addition of GOPS. Samples (w/ or w/o GOPS) were immersed in water for up to 7 days. These samples have a width of 1 cm and a length of 0.5 cm.

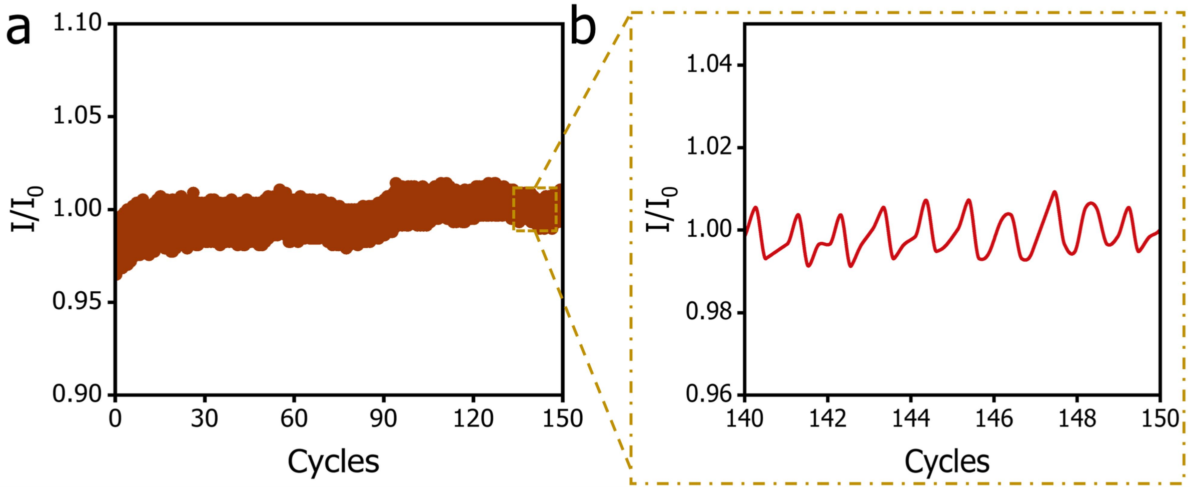

Figure S13. The cyclic stability of the electromechanical performance of PEDOT: PSS film on TPU. (a) The current variation between 0% and 30% strain is recorded for 150 strain cycles and (b) a zoom-in image for the strain cycles between 140 and 150. Noted that I_0 denotes the initial current, and I denote the real-time recorded current.

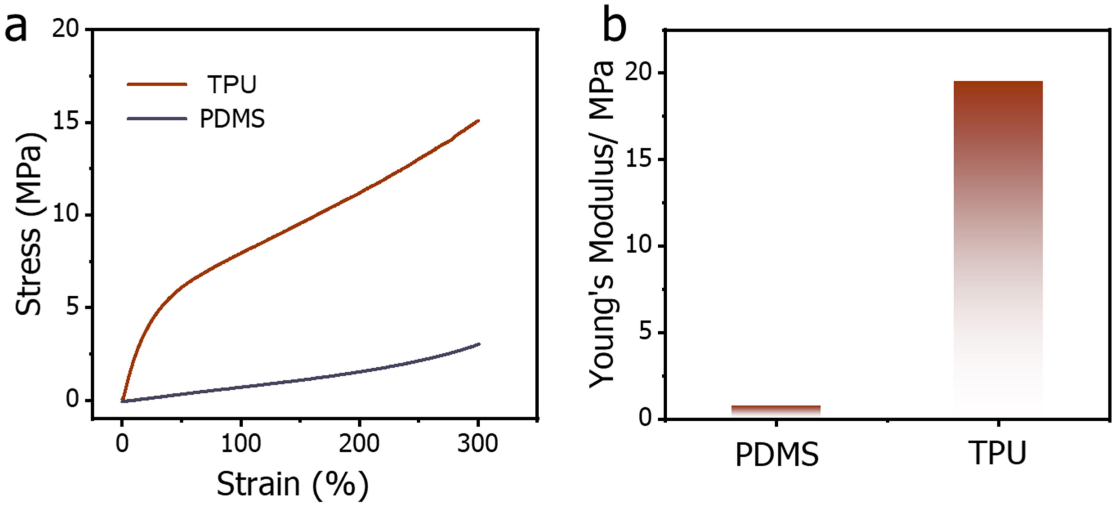

Figure S14. Mechanical profiles of TPU and PDMS elastomers. (a) the stress-strain curve of TPU and PDMS elastomers. (b) The comparison of Young's Modulus of PDMS and TPU. The data was extracted directly from (a).

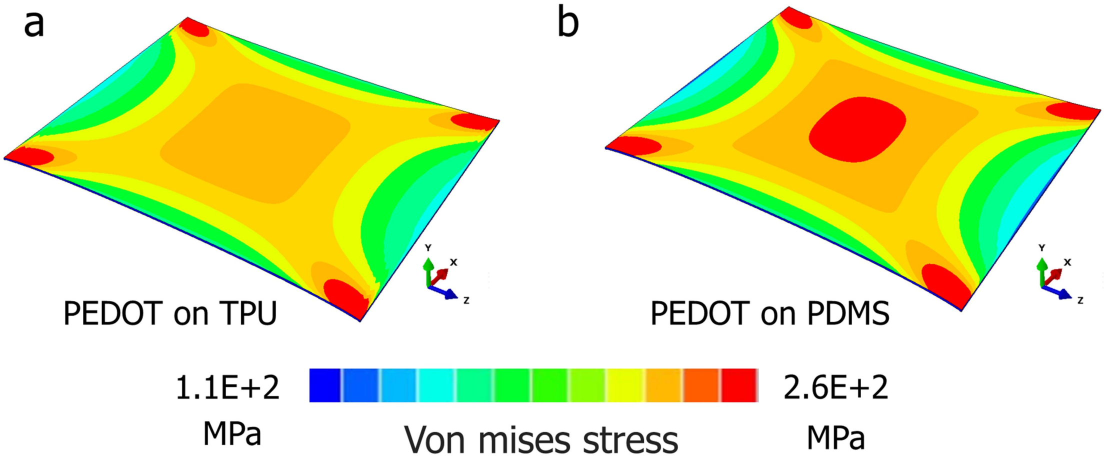

Figure S15. Finite element analysis (FEA). The stress distribution of PEDOT: PSS films on (a) TPU and (b) PDMS substrates at 30 % strain.

Table S1. Benchmarking the performance of stretchable OECTs reported so far.

Literature	Substrate	Channel composition*	Dimension (mm)		On/off ratio	Mobility (cm^2 V^{-1} s^{-1})	Tolerance strain (%)
			W	L			
Ref. S1[2]	PDMS	PEDOT: PSS/EG/ DBSA/GOPS/Capstone FS-30	0.63	0.13	3	N/A	140
Ref. S2[3]	PDMS	PEDOT: PSS/EG/ DBSA/GOPS/Capstone FS-30	0.4	0.25	~3	N/A	38
Ref. S3[4]	PDMS	PEDOT: PSS/Gly/ DBSA/GOPS/ Capstone FS-30	4	0.01	2-10	N/A	30
Ref. S4[5]	PDMS	PEDOT: PSS/Gly/ DBSA/GOPS/ Capstone FS-30	4	0.01	7-150	N/A	30
Ref. S5[6]	Skin	PEDOT: PSS/Gly/ DBSA/GOPS/ Capstone FS-30	10	1	50	N/A	5
Ref. S6[7]	SEBS	PEDOT: PSS/ [EMIM][Cl] /GOPS	1	0.25	~500	N/A	N/A
Ref. S7[8]	PDMS	PEDOT: PSS/PEG/Gly/ DBSA/ Capstone FS-30	8	2	50-160	N/A	45
Ref. S8[9]	PDMS	PEDOT: PSS/xylitol/EG/ GOPS/ Capstone FS-30	N/A	N/A	170	1.16	30
Ref. S9[10]	Acrylic Elastomer	PEDOT: PSS/EG/DBSA/GOPS	0.05	0.05	~20	N/A	30
Ref. S10[11]	PDMS	PEDOT: Tos/ PEO	2	15	45	N/A	50
Ref. S11*	PDMS	PEDOT: PSS/Gly/ DBSA/GOPS	5	0.1	10-300	0.08	<30
**This work**	**TPU**	**PEDOT: PSS/Gly/ DBSA/GOPS**	**5**	**0.1**	**8000**	**1.11**	**≥50**

*** Note:** EG, Gly respectively denote the ethylene glycol and glycerol that serves as conductivity enhancers; DBSA denotes the dodecylbenzene sulfonic acid surfactant; GOPS denotes the (3-glycidyloxypropyl) trimethoxy silane cross-linker;[12] Capstone (Capstone FS-30) is used to improve the stretchability of PEDOT: PSS film[13]; [EMIM][Cl] refers to 1-ethyl-3-methylimidazolium chloride, which could improve the processability and conformability of PEDOT: PSS;[7] Xylitol is added to improve the stretchability and hole mobility.[9] PEDOT: Tos was reported fabricated through vaper phase polymerization.[11] Tos denotes tosylate. PEO denotes poly (ethylene oxide). N/A denotes such parameter is not available from the published literature. Ref. 11 refers to the stretchable OECT on PDMS measured in this work.

Reference

[1] D. A. Bernards, G. G. Malliaras, *Adv. Funct. Mater*. **2007**, 17, 3538.

[2] N. Matsuhisa, Y. Jiang, Z. Liu, G. Chen, C. Wan, Y. Kim, J. Kang, H. Tran, H. C. Wu, I. You, *Adv. Electron. Mater.* **2019**, 5, 1900347.

[3] B. Marchiori, R. Delattre, S. Hannah, S. Blayac, M. Ramuz, *Sci. Rep.* **2018**, 8, 8477.

[4] S. Zhang, E. Hubis, G. Tomasello, G. Soliveri, P. Kumar, F. Cicoira, *Chem. Mater.* **2017**, 29, 3126.

[5] S. Zhang, Y. Li, G. Tomasello, M. Anthonisen, X. Li, M. Mazzeo, A. Genco, P. Grutter, F. Cicoira, *Adv. Electron. Mater.* **2019**, 5, 1900191.

[6] S. Zhang, H. Ling, Y. Chen, Q. Cui, J. Ni, X. Wang, M. C. Hartel, X. Meng, K. Lee, J. Lee, W. Sun, H. Lin, S. Emaminejad, S. Ahadian, N. Ashammakhi, M. R. Dokmeci, A. Khademhosseini, *Adv. Funct. Mater.* **2020**, 30, 1906016.

[7] X. Wu, A. Surendran, M. Moser, S. Chen, B. T. Muhammad, I. P. Maria, I. McCulloch, W. L. Leong, *ACS Appl. Mater. Interfaces* **2020**, 12, 20757.

[8] Y. Li, S. Zhang, X. Li, V. R. N. Unnava, F. Cicoira, *Flex. Print. Electron.* **2019**, 4, 044004.

[9] T. D. Nguyen, T. Q. Trung, Y. Lee, N.-E. Lee, *Adv. Eng. Mater.* **2021**, 2100918.

[10] M. Lerond, A. Subramanian, W. G. Skene, F. Cicoira, *Front. Phys.* **2021**, 9.

[11] H. Lee, S. Lee, W. Lee, T. Yokota, K. Fukuda, T. Someya, *Adv. Funct. Mater.* **2019**, 29, 1906982.

[12] S. Zhang, P. Kumar, A. S. Nouas, L. Fontaine, H. Tang, F. Cicoira, *APL Mater.* **2015**, 3, 014911.

[13] D. J. Lipomi, J. A. Lee, M. Vosgueritchian, B. C.-K. Tee, J. A. Bolander, Z. Bao, *Chem. Mater.* **2012**, 24, 373.
